# Supplementary material for: Radiotherapy-Induced High Neutrophil-to-Lymphocyte Ratio is a Negative Prognostic Factor in Patients with Breast Cancer
Source: Cancers (Basel). 2020 Jul 14;12(7):1896. doi: 10.3390/cancers12071896 (PMC7409084; doi:10.3390/cancers12071896)
Supplement: Supplementary file 1 [file cancers-12-01896-s001.zip › cancers-877288-supplementary-final/Supplementary figure.docx]

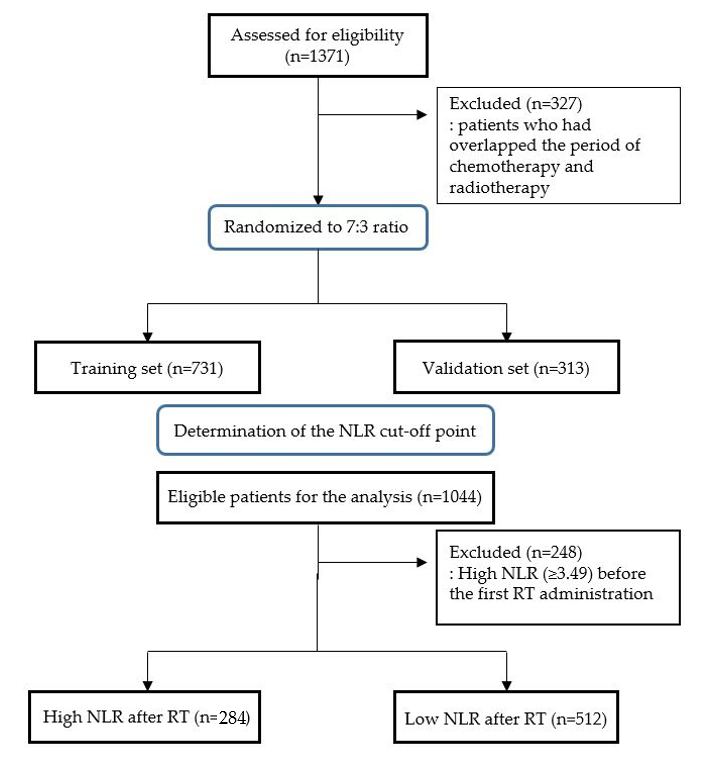


**Figure S1.** Consort diagram of study. Of the 1371 patients enrolled in this study, 327 patients were excluded because they received RT during chemotherapy. 1044 patients were divided into training and validation sets, in a 7:3 ratio (731 and 313, respectively). The NLR cut-off point was determined to be 3.49. A total of 248 patients were excluded due to high NLR before the first RT administration (T1). Of the 284 patients had RT-induced high NLR, and 512 patients had low NLR after RT (T2).


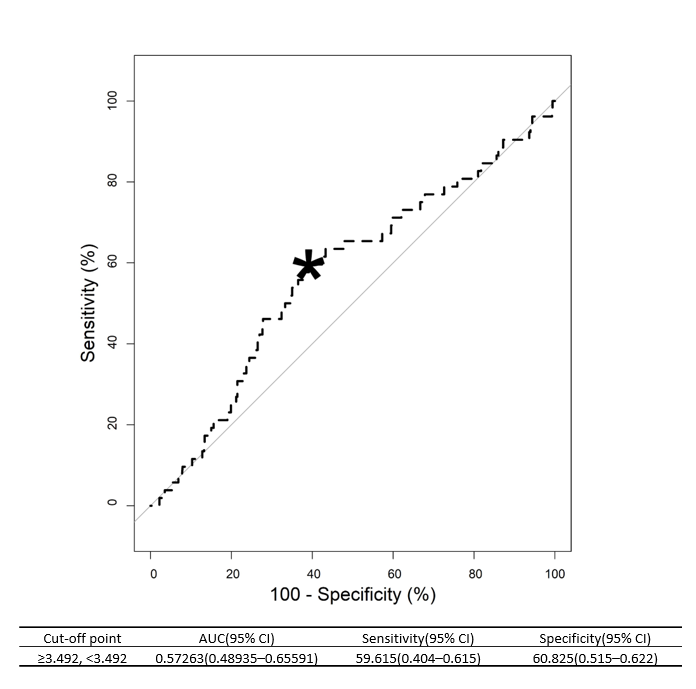


**Figure S2.** Determination of the NLR cut-off point using the Youden-index. All patients were randomly divided into training and validation sets (7:3). When NLR was 3.49, sum of sensitivity and specificity in the training set (*n* = 731) was the maximum. When the cut-off point of NLR was 3.49, the AUC, sensitivity, and specificity were 0.57263, 59.6%, and 60.8%, respectively. AUC, area under the curve; CI, confidence interval.


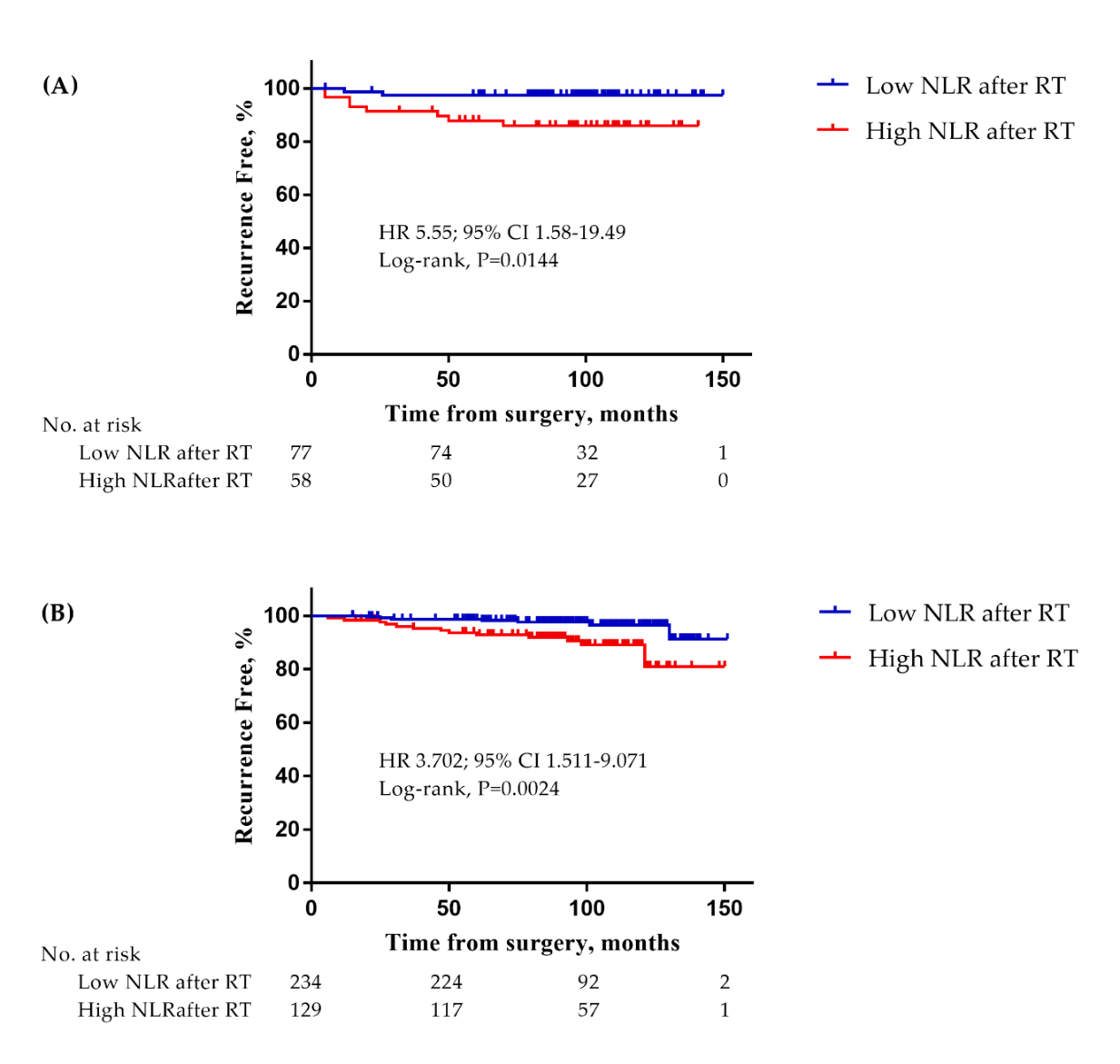


**Figure S3.** Kaplan-Meier survival curves of patients (a) taking SERM, and (b) not taking SERM. (a) patients with high NLR after RT showed a lower RFS (HR 5.55; 95% CI 1.58–19.49, P = 0.0144), (b) patients with high NLR after RT showed a lower RFS (HR 3.702; 95% CI 1.511–9.071, P = 0.0024).
