# Supplementary material for: Radiotherapy-Induced High Neutrophil-to-Lymphocyte Ratio is a Negative Prognostic Factor in Patients with Breast Cancer
Source: Cancers (Basel). 2020 Jul 14;12(7):1896. doi: 10.3390/cancers12071896 (PMC7409084; doi:10.3390/cancers12071896)
Supplement: Supplementary file 1 [file cancers-12-01896-s001.zip › cancers-877288-supplementary-final/Supplementary table.docx]

**Table S1.** Distribution of chemotherapy regimen according to NLR after RT in patients who received chemotherapy.

| **Variables** | **High NLR/with Chemotherapy, *n* = 205 (%)** | **Low NLR/with Chemotherapy, *n* = 145(%)** | ***p* Value** |
| --- | --- | --- | --- |
| Chemotherapy regimen |  |  | 0.661 |
| Anthracycline | 98 (47.8) | 63 (43.4) |  |
| Anthracycline based + taxane | 45 (22.0) | 37 (25.5) |  |
| Other | 62 (30.2) | 45 (31.0) |  |

**Table S2.** The HRs and 95% CIs for overall survival (OS) according to RT-induced high NLR.

| **Variables** | **Univariate Analysis** | | **Multivariate Analysis** | |
| --- | --- | --- | --- | --- |
|  | **HRs (95% CIs)** | ***p* Value** | **HRs (95% CIs)** | ***p* Value** |
| Age, year |  | 0.339 |  |  |
| >50 | 1 |  |  |  |
| ≤50 | 0.718(0.365–1.414) |  |  |  |
| NG |  | 0.002 |  |  |
| I, II | 1 |  |  |  |
| III | 3.075(1.503–6.290) |  |  |  |
| HG |  | 0.021 |  |  |
| I, II | 1 |  |  |  |
| III | 2.240(1.131–4.437) |  |  |  |
| ER |  | 0.004 |  | <0.001 |
| Negative | 1 |  | 1 |  |
| Positive | 0.374(0.190–0.736) |  | 0.261(0.125–0.543) |  |
| PR |  | 0.072 |  |  |
| Negative | 1 |  |  |  |
| Positive | 0.539(0.275–1.057) |  |  |  |
| HER2 |  | 0.038 |  | 0.044 |
| Negative | 1 |  | 1 |  |
| Positive | 2.049(1.042–4.032) |  | 2.141(1.021–4.491) |  |
| Tumor size, cm |  | 0.015 |  |  |
| ≤2 | 1 |  |  |  |
| >2 | 2.499(1.194–5.227) |  |  |  |
| LN metastasis |  | <0.001 |  | 0.005 |
| Negative | 1 |  | 1 |  |
| Positive | 3.824(1.906–7.674) |  | 3.002(1.388–6.491) |  |
| Subtype |  | 0.040 |  |  |
| Luminal/HER2(-) | 1 |  |  |  |
| HER2(+) | 2.580(1.188–5.605) |  |  |  |
| TNBC | 2.199(0.799–6.052) |  |  |  |
| RT-induced high NLR |  | 0.005 |  | 0.027 |
| <3.49 | 1 |  | 1 |  |
| ≥3.49 | 2.643(1.335–5.236) |  | 2.394(1.106–5.181) |  |
| Regional node irradiation |  | <0.001 |  |  |
| Done | 1 |  |  |  |
| Not done | 3.515(1.766–6.995) |  |  |  |
| Chemotherapy |  | 0.001 |  |  |
| Done | 1 |  |  |  |
| Not done/unknown | 3.471(1.648–7.309) |  |  |  |

Abbreviations: NG, nuclear grade; HG, histologic grade; ER, estrogen receptor; PR, progesterone receptor; HER-2, human epidermal growth factor receptor-2; LN, lymph node; TNBC, triple negative breast cancer; RT, radiotherapy; NLR, neutrophil to lymphocyte ratio.
